# *F. novicida*-Infected *A. castellanii* Does Not Enhance Bacterial Virulence in Mice

**DOI:** 10.3389/fcimb.2016.00056

**Published:** 2016-05-18

**Authors:** Mateja Ozanic, Ivana Gobin, Martin Brezovec, Valentina Marecic, Zlatko Trobonjaca, Yousef Abu Kwaik, Marina Santic

**Affiliations:** ^1^Department of Microbiology and Parasitology, Faculty of Medicine, University of RijekaRijeka, Croatia; ^2^Department of Physiology and Immunology, Faculty of Medicine, University of RijekaRijeka, Croatia; ^3^Department of Microbiology and Immunology and Center for Predictive Medicine, College of Medicine, University of LouisvilleLouisville, KY, USA

**Keywords:** *Francisella*, amoeba, mice, tularemia, pathogenesis

## Abstract

*Francisella tularensis* is a facultative intracellular bacterium that causes tularemia in humans and animals. Epidemiology of tularemia worldwide is often associated with water-borne transmission, which includes mosquitoes and amoebae as the potential host reservoirs of the bacteria in water environment. *In vitro* studies showed intracellular replication of *F. tularensis* within *Acanthamoeba castellanii* and *Hartmanella vermiformis* cells. While infection of amoeba by *Legionella pneumophila* has been shown to enhance infectivity of *L. pneumophila* the role of *F. tularensis-*infected protozoa in the pathogenesis of tularemia is not known. We used 6 h coculture of *A. castellanii* and *F. novicida* for investigation of the effect of inhaled amoeba on the pathogenesis of tularemia on *in vivo* model. Balb/c mice were infected intratracheally with *F. novicida* or with *F. novicida-*infected *A. castellanii*. Surprisingly, infection with *F. novicida-*infected *A. castellanii* did not lead to bronchopneumonia in Balb/c mice, and *Francisella* did not disseminate into the liver and spleen. Upon inhalation, *F. novicida* infects a variety of host cells, though neutrophils are the predominant cells early during infection in the lung infiltrates of pulmonary tularemia. The numbers of neutrophils in the lungs of Balb/c mice were significantly lower in the infection of mice with *F. novicida*-infected *A. castellanii* in comparison to group of mice infected only with *F. novicida*. These results demonstrate that following inoculation of mice with *F. novicida*-infected *A. castellanii*, mice did not develop tularemia.

## Introduction

*Francisella tularensis* is a gram negative bacterium and causative agent of zoonotic disease, tularemia. Five species of the genus *Francisella* has been recognized: *F. tularensis, F. philomiragia, F. hispaniensis, F. noatunensis*, and *F. novicida* (Sjödin et al., 2012; Kingry and Petersen, 2014). Tularemia in humans is mostly caused by two subspecies of *F. tularensis, tularensis* (Type A) and *holarctica* (Type B). *F. novicida* U112 is avirulent in immunocompetent humans but is very virulent in experimental mice, only few bacteria cause disease and death, similar to *Francisella tularensis* subsp. *tularensis* (Sjödin et al., 2012). The most common way of transmission of the disease is by exposure to infected arthropod vectors, or by handling, ingesting, or inhaling infectious materials. Aerosol transmission of *F. tularensis* cause most severe tularemia leading to mortality rates up to 30% with the *F. tularensis* subsp. *tularensis* (Dienst, 1963).

*F. tularensis* has been found in many animal species, including fish, birds, amphibians, rabbits, squirrels, hares, voles, ticks, and flies (Sjöstedt, 2007; Akimana and Kwaik, 2011). *In vitro* studies showed that *F. tularensis* can survive and grow within *A. castellanii* and *H. vermiformis* cells (El-Etr et al., 2009; Santic et al., 2011) as well as within amoebal cysts (El-Etr et al., 2009). The isolation of *F. tularensis* subsp. *holarctica* from rivers, lakes, streams and ponds (Willke et al., 2009; Broman et al., 2011) supports the hypothesis that protozoa may serve as a reservoir for *F. tularensis* in nature (Willke et al., 2009; Broman et al., 2011). Very little is known about the *F. tularensis*-amoeba interaction. Free-living amoebae such as *Acanthamoeba* and *Hartmannella* are environmental hosts of several intracellular pathogens such as *Legionella, Chlamydia*, and *Mycobacterium* (Thomas et al., 2008; Jacquier et al., 2013). *L. pneumophila* is an intracellular gram-negative bacterium, ubiquitous in the aquatic environment and important causative agent of community-acquired and nosocomial bacterial pneumonia. The bacterium enters the human body via inhalation of aerosol droplets. Once in the lungs, *L. pneumophila* invades and replicate mainly in alveolar macrophages (Richards et al., 2013). The pathogenesis of legionellosis depends also on prior adaptation of *L. pneumophila* in the natural water environment (Richards et al., 2013). In freshwater, *Legionella* survive and replicate within free-living protozoa including ciliates *Tetrahymena* and *Cyclidium* spp. as well as amoeba species belonging to *Acanthamoeba, Hartmanella, Valkampfia, Naegleria*, and *Dictyostelium* (Barbaree et al., 1986; Cianciotto and Fields, 1992; Declerck et al., 2007a,b; Dey et al., 2009; Bozzaro and Eichinger, 2011; Tyson et al., 2014). It has been shown that *Legionellae* interact with their protozoan hosts and mammalian cells in a similar way (Harb et al., 2000; Brüggemann et al., 2006). The amoeba-grown *Legionella* were found to enter the macrophages at a higher frequency than agar-grown legionela (Cirillo et al., 1994). In addition, it has been shown that growth of *L. pneumophila* in the lungs of A/J mice is potentiated by *L. pneumophila*-infected *H. vermiformis* (Brieland et al., 1996). Similarly, *A. castellanii*-grown *L. pneumophila* is more infectious than agar grown bacteria (Cirillo et al., 1999). These data show that coincident inhalation of protozoa harboring *L. pneumophila* enhances the severity of Legionnaire's disease. Our previous results showed a different intracellular lifestyle of *F. novicida* within *H. vermiformis* in comparison to mammalian cells (Santic et al., 2011). While in mammalian cells cytosolic location of bacteria after escape from the vacuole is a crucial step in productive intracellular replication, in amoeba cells the bacteria are enclosed in the vacuole where they replicate (Abd et al., 2003; Santic et al., 2011).

*F. tularensis* does not induce a classical pulmonary pro-inflammatory immune response (Bosio et al., 2007; Chase et al., 2009; Allen, 2013). The flow cytometry analysis of *F. tularensis* infected mouse lung cells indicates that neutrophils are the predominant infected cells by day 3 after infection (Hall et al., 2008; Allen, 2013). In addition, it has been shown that neutrophils are crucial for early host defense against systemic, but not respiratory LVS (live vaccine strain) *F. tularensis* infection in mice (Conlan et al., 2002). It seems that macrophages are central to the innate response to infection while neutrophils plays a role through initiating immune cell infiltration (Cowley and Elkins, 2011; Allen, 2013). However, little is known about the role of neutrophils in pathogenesis of the disease.

Tularemia is most deadly in the pneumonic form. After inhalation of contaminated dust, often by farming activities or landscaping respiratory tularemia could occur. During the respiratory form of disease, pneumonia may not always be present but may be present in other forms of tularemia (Santic et al., 2007b; Moskowitz and Wiener-Kronish, 2010). *Francisella* replicate at the initial site of entry, and then spread to the lymph nodes, liver and spleen (Forestal et al., 2007; Santic et al., 2007b; Sharma et al., 2011). It has been speculated that *F. tularensis* persists in water between tularemia outbreaks (Broman et al., 2011). We have previously demonstrated that Balb/c mice develop replicative *F. novicida* lung infection in response to intratracheal inoculation with virulent *F. novicida*, with maximal intrapulmonary growth of *F. novicida* at 72 h postinoculation (Santic et al., 2007b). In the current study, we tested the hypothesis that, similar to *L. pneumophila*, inhaled *F. tularensis*-infected protozoon may constitute an infectious particle for tularemia. We showed the bacterium enclosed in the vacuole of amoeba cells within the lungs. The results demonstrate that infection of *F. novicida-*infected *A. castellanii* organism does not initiate neither pulmonary nor systemic tularemia in Balb/c mice. The recruitment of polymorphonuclear cells is not evident during coinfection of Balb/c mice with *F. novicida*-infected *A. castellanii.* These results demonstrate that in contrast to *L. pneumophila*, inhaled protozoa do not serve as a vehicle in the pathogenesis of respiratory murine tularemia caused by *F. novicida*.

## Materials and methods

### Bacteria and amoeba cultures

The wild type *F. novicida* strain U112 (Fn) and *L. pneumophila* strain AA100 (Lpn) were grown on buffered-charcoal yeast extract (BCYE) agar plates as have been described previously (Santic et al., 2005b, 2007a). *A. castellanii* was obtained from the American Type Culture Collection, 30234. The amoebae were grown in medium 30234 at 25°C, as described elsewhere (Pedersen et al., 2001; El-Etr et al., 2009; Santic et al., 2011). For preparation of the inoculums, *A. castellanii* were collected from the culture flasks, centrifuged (350xg, 30 min), resuspended in PBS, counted in hemocytometer (Neubauer chamber), washed once in phosphate-buffered saline (PBS) and suspended in PBS at 10^5^ cells per ml.

### *In vitro* infection assay

To examine the virulence of amoeba-grown bacteria in mice, we used *L. pneumophila* as a control by the methods described previously (Cirillo et al., 1994; Brieland et al., 1997b).

For preparation of *F. novicida*-infected *A. castellanii*, confluent monolayers of *A. castellanii* were inoculated with *F. novicida* at a multiplicity of infection of 10 for 30 min. After 30 min, the monolayers were washed to remove nonadherent bacteria and incubated in media containing gentamicin (100 μg/ml) for 1 h to kill extracellular bacteria. Antibiotic-containing medium was subsequently removed and replaced with antibiotic-free medium, and *F. novicida* infected-amoeba monolayers were incubated for further 6 h period, to reach the number of bacterium within the amoeba cells of 10^4^ cfu/ml. One hour prior to harvest, the monolayers were treated again with gentamicin (100 μg/ml). *F. novicida*-infected amoebae were subsequently removed from the flasks, resuspended in PBS and used *in vivo* within 30 min of preparation. To determine the number of bacteria, amoeba was lysed with Triton × 100 (0.1%) for 10 min, washed in PBS, measured by spectophotmetry and plated on BCYE agar, respectively. Results of our experiment showed that *F. novicida* replicated in amoeba resulting in number of bacteria 10^4^ cfu/ml at 6 h after infection. The *in vivo* virulence of *Francisella* after growing in amoeba was determined as well. The 6 h coculture of *F. novicida* and *A. castellanii* (MOI 10) was centrifuged for 30 min at 350 × g to pellet the bacteria and amoeba. The pellet was suspended in 1 ml of distilled water for 10 min and passed through a 27-gauge syringe three times to lyse remaining amoebae as described. To remove any remaining amoebae, we centrifuged this preparation for 1 min at 150 × g and transferred the supernatant to the new tube. The *Francisella* (10^3^ cfu per mouse) and/or *Legionella* suspension (10^5^ cfu per mouse) were used *in vivo*.

### Mice infection

Female pathogen-free Balb/c and A/J mice, 8–9 weeks of age, were used in all experiments. Mice were housed in specific pathogen free conditions within our animal care facility according to standard guidelines, and the use of animals for infection was approved by the institutional IACUC.

The mice were anesthetized by intraperitoneal (i.p.) injection of ketamine (2.5 mg per mouse). The incision was made through the skin of the ventral neck, the trachea was isolated and 50 μl of the bacterial suspension in sterile saline was inoculated using a 26-gauge needle followed by 10–20 μl of air by intratracheal infection (i.t.). A/J mice were inoculated with *L. pneumophila* (10^5^ cfu per mouse) and/or *L. pneumophila*-infected *A. castellanii* (MOI 10, 10^6^ amoebae containing 10^5^ bacteria, harvested after 6 h of coculture). Balb/c mice were inoculated with *F. novicida* (10^3^ cfu per mouse), *A. castellanii* (10^4^ cells per mouse) and/or with *F. novicida*-infected *A. castellanii* (MOI 10, harvested after 6 h of coculture). Control animals were inoculated with saline only. The skin incision was surgically closed. At 2, 24, 48, and 72 h postinoculation, the mice were euthanized, the lung, liver and spleen were removed and homogenized, and *F. novicida* cfu were determined by culture of tissue homogenates on BCYE agar. The cfu of *L. pneumophila* were determined in the lung homogenates.

For mortality, 10 mice per group were infected either with *F. novicida, A. castellanii*, and/or *F. novicida*-infected *A. castellanii* as described above. Mice were observed daily during 15 days period.

### Histopathology studies

The histological changes in the lungs of Balb/c mice in response to infection were assessed by light microscopy as described previously (Santic et al., 2007b). At 2, 24, 48, and 72 h after inoculation, the mice were humanely sacrificed. Before organ removal, the pulmonary vasculature was perfused with 10 ml of saline containing 5 μM EDTA, via the right ventricle. The excised organs were fixed in 10% neutral formalin for 24 h, dehydrated and embedded in paraffin. Sections (5 μm) were cut, stained with haematoxylin and eosin (H&E), and analyzed by light microscopy. On average of 10 0.2 μm thick serial sections of each image were captured and stored for further analyses. Twenty random high-powered fields (HPFs) were assessed to grade inflammation severity including alveolar and bronchial damage as well as percentage of parenchyma involved. The histology assessment included the number of the mononuclear cells and percent of parenchyma involved by using modification of double-blind scoring method at a magnification of 40x, as described previously (De Simone et al., 2014). The inflammation process was graded normal (score of 0) when there were 0–19 monocular cells infiltrates per HPF with no alveolar and bronchial involvement, mild (score of 1) for 20–49 cells per HPF including mild damage of alveolar and bronchial regions, moderate (score of 2) for 50–99 cells per HPF with moderate alveolar and bronchial inflammation, or severe (score of 3) for 100–200 mononuclear cells per HPF with severe effacement of alveolar and bronchial regions. The murine lung section was examined in sagittal direction and percent of parenchyma involved was scored as 0 when no area was compromised. The involvement of the parenchyma was scored as 1 when up to 25% of the total area was occupied by inflammatory exudate; was scored as 2 when 26–50% of parenchyma area was occupied with inflammatory cells, 3 if comprised more than 51%. The total histology score was calculated as an average of individual criteria scores. The uninfected tissue was used as a baseline score, ^*^P < 0.05 and ^**^P < 0.001. In addition, statistically significant differences between *F. novicida* and *F. novicida*-infected *A. castellanii* groups are marked by ^⋆^*p* < 0.05. The bar represents the median score in each group.

### Inflammatory cell recruitment into the lung

Mice were intratracheally inoculated with *F. novicida, A. castelannii* and/or with *F. novicida*-infected *A. castellanii*. All mice were subsequently processed and analyzed individually. At 24 h after infection, mice were humanely sacrificed. The lungs were excised, minced, and incubated in RPMI 1640 medium containing 5% fetal calf serum, 1 mg of collagenase A (Sigma Chemical Company, St. Louis, Mo) per ml, and 20 μl DNase per ml (Sigma Chemical Company, St. Louis, Mo.) for 60 min at 37°C in shaking incubator. The cells were further disaggregated by drawing the lung homogenate repeatedly through a 10-ml syringe 20–30 times prior to pelleting of the cells by centrifugation. Cells were isolated using Percoll (Fluka) gradient. Isolated cells were resuspended in PBS supplemented with 2% FCS and 0.03% NaN_3_ and blocked with purified rat anti-mouse CD16/CD32 (BD Pharmingen). The cells were phenotyped using monoclonal antibodies specific for the following leukocyte surface antigens: anti-CD11b (BD Pharmingen) and anti-GR-1 (Miltenyi Biotec). Lymphocyte and macrophages were gated according to their size and granularity defined in the forward light scatter (FSC) and side light scatter (SSC) plot. Neutrophils were gated as CD11b^+^ and GR-1^+^ cells while mononuclear cells were gated as CD11b^+^ and GR-1^−^ cells. The immunofluorescence analysis was performed by BD FACS Calibur™ flow cytometer using CellQuest™ software.

### Electron microscopy

For electron microscopy, at 2 and 24 h after infection lung tissues of Balb/c mice were removed and fixed in 2.5% gluteraldehyde (infection procedure is described above). Briefly, lungs were post fixed by immersion in 2% osmium tetroxide in 0.1 M sodium Sorenson's buffer for 1 h, followed by dehydration in acetone, infiltration and embedding in Epon 12 epoxy resin. Sections (0.5 μm) were stained with toluidine blue and scanned by light microscopy to define areas containing bacteria for ultrastructural examination. Ultrathin sections (0.1 μm) were then cut, stained with uranyl acetate and lead citrate, and examined in Philips transmission electron. The integrity of phagosomal membrane was determined by electron microscopy counting at least 100 bacteria for each sample and using following criteria: (a) cytosolic localization of bacteria, (b) vacuolar localization of bacteria- intact vacuoles.

### Statistics

Statistical analyses were performed with GraphPad Prizm version 6.0 software. Bacterial burdens, cell populations and histopathology scoring were compared by *t*-test. Survival curves were compared by the log-rank Mantel-Cox test. ^*^P < 0.05 and ^**^P < 0.001 were accepted as significantly different.

### Ethics statement

All the experimental procedures were in compliance with National guidelines and were approved by the Institutional Animal Care and Use committee (IACUC) at Faculty of Medicine, University of Rijeka.

## Results

### The survival and growth of *F. novicida* in the organs of Balb/c mice inoculated with *F. novicida*-infected *A. castellanii*

It has been shown that *F. novicida* replicates in different phagocytic and non phagocytic cells. Our and other studies have shown that *F. tularensis* subsp. *holarctica, tularensis*, and *F. novicida* survive and replicate in *A. castellanii* while *F. novicida* is able to replicate in *H. vermiformis* as well (Abd et al., 2003; El-Etr et al., 2009; Santic et al., 2011). *F. novicida*, although avirulent for humans, is highly virulent in Balb/c and C57Bl/6 mice causing severe tularemia (Kieffer et al., 2003; Lauriano et al., 2004; Pammit et al., 2004; Shen et al., 2004; Mares et al., 2010). The severity of the disease is dependent on bacterial strain and the route of infection (Conlan et al., 2011). Our previous results showed that the dissemination of *Francisella* in Balb/c mice following intratracheal infection with the wild-type strain of *F. novicida* is similar to that previously reported for intranasal or aerosol infection (Santic et al., 2007b).

**Figure 1:**
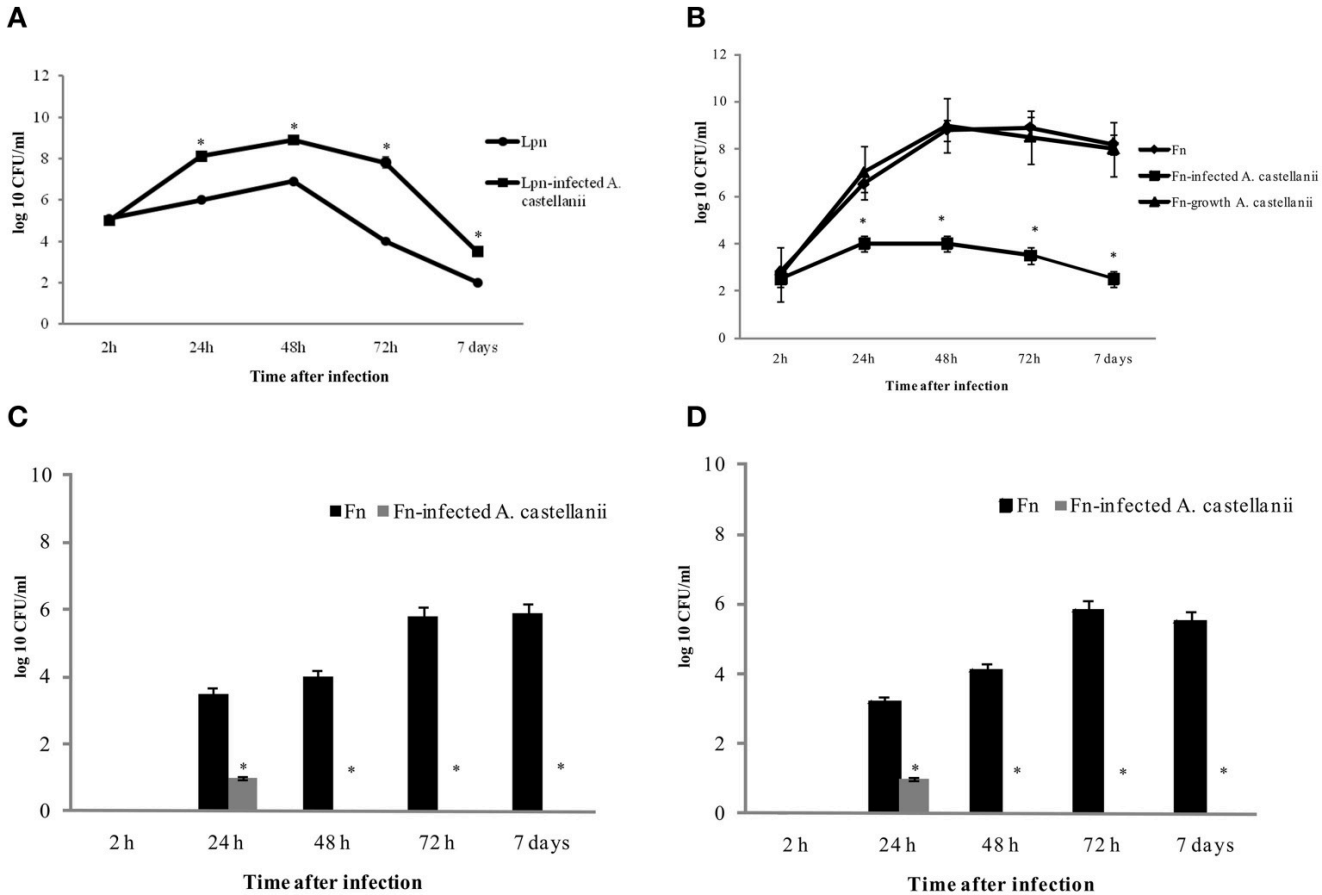
**Growth kinetics of *L. pneumophila* and *L. pneumophila*-infected *A. castellanii* in lungs of A/J mice (A); *F. novicida* and *F. novicida*-infected *A. castellanii* in lungs (B), liver (C), and spleen (D) of Balb/c mice**. At different time points after intratracheal infection of mice, organs were removed for determination of the number of bacteria (cfu) by plating serial dilutions on agar plates. The error bars represent standard deviations of triplicate samples and the results shown are representative of three independent experiments, ^*^p < 0.05.

We used previously established murine model of *L. pneumophila*-infected amoeba as control for this study (Cirillo et al., 1994; Brieland et al., 1997b). A/J mice were inoculated with *L. pneumophila* (10^5^ cfu per mouse) and/or *L. pneumophila*-infected *A. castellanii* (MOI 10, 10^6^ amoebae containing 10^5^ bacteria, harvested after 6 h of coculture). Consistent with previous studies, *L. pneumophila* replicated more robustly in the lungs of mice that were infected with *L. pneumophila*–infected A. *castellani* (Figure 1A). In contrast, intrapulmonary growth of *L. pneumophila* was significantly lower in A/J mice inoculated with *L. pneumophila* alone (Figure 1A) (*t*-test, *p* < 0.05).

Balb/c mice were inoculated intratracheally with *F. novicida* (10^3^ bacteria per mouse), *A. castellani* (10^4^ cells per mouse) and/or *F. novicida*-infected *A. castellanii* (10^3^ bacteria per mouse in 10^4^ cells per mouse- determined after 6 h culture). We also compared the virulence of intracellular *F. novicida* grown in amoeba for 6 h. The amoeba were lysed, and 10^3^ of purified intracellular *F. novicida* were inoculated intratracheally in the Balb/c mice. As a control one group of mice were infected with *A. castellanii* alone (10^4^ cells per mouse).

At 2, 24, 48, and 72 h postinoculation, the mice were euthanized, the lungs, liver and spleen were excised and homogenized, and the numbers of *F. novicida* cfu per organs homogenate were compared. Our results showed that when mice were inoculated with *F. novicida* alone, bacteria proliferated robustly in the lungs of mice with the peak of infection at 48 and 72 h, where the number of bacteria reached almost 10^9^ cfu (Figure 1B). In contrast the number of bacteria in the lungs of *F. novicida*-infected *A. castellani* mice reached only around 10^4^ cfu at 48 h after infection (*t-*test, *p* < 0.05) (Figure 1B). At all time points examined there was a significant difference (*t*-test, *p* < 0.05) in the cfu of bacteria in the lungs of infected mice between these two experimental groups (Figure 1B). In the group of mice infected with purified *F. novicida* after growing in amoeba cells for 6 h we did not find any differences in intracellular replication of bacteria in lungs of mice compared to mice infected with BCYE-grown *F. novicida* (Figure 1B; *t*-test, *p* was 0.05).

The dissemination of *F. novicida* to the liver and spleen after infection *F. novicida*-infected *A. castellanii* was also assessed. In the group of mice infected with *F. novicida* alone the number of bacteria that reached the spleen and liver were up to 10^6^ cfu/ ml at 72 h after infection (Figures 1C,D). In contrast, only 10 bacteria reached the liver and spleen after infection of mice with *F. novicida*-infected *A. castellani* (Figures 1C,D; *t*-test, *p* < 0.05).

### The *Francisella*-amoeba coinfection do not enhance the mortality of mice

**Figure 2:**
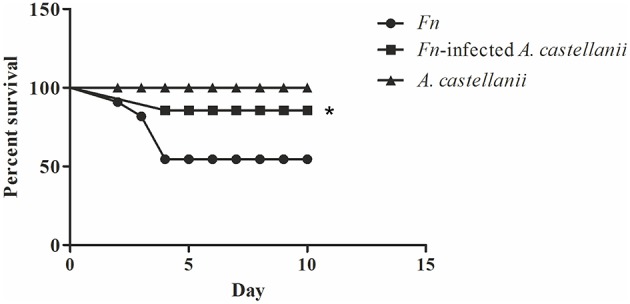
**Survival of Balb/c mice**. Balb/c mice inoculated intratracheally with *F. novicida, A. castellanii*, and/or *F. novicida*-infected *A. castellanii* were observed twice daily for 15 days for clinical signs of illness or survival. Results represent combined results of two separate experiments and 10 animals per treatment group, ^*^p < 0.05.

There is little differences in susceptibility to tularemia infections depending on mice strain (Fritz et al., 2014) but Balb/c mice were most commonly used in experimental tularemia. We infected Balb/c mice intratracheally with *F. novicida, A. castellanii*, and/or *F. novicida*-infected *A. castellanii*. The dose of 1 × 10^3^ cfu per mouse was used for infection with *F. novicida* which is sufficient to cause mortality in 50% of mice. After the infection, mice were observed during 15 days (Figure 2).

We observed that mice infected with *F. novicida* exhibited clinical signs of infection. Symptoms persisted and worsened, and 40% of mice died between 3 and 4 days post infection with *F. novicida* (Figure 2).

The symptoms of the disease in the group of *F. novicida*-infected *A. castellanii* mice were much milder compared the group of mice infected with *F. novicida* alone. In this group of mice, 90 % of mice survived the infection (Figure 2) (*Mantel-Cox*-test, *p* < 0.05).

The group of mice infected with *A. castellanii* alone did not show any symptoms of the disease and this group were similar to uninfected mice. The infected Balb/c mice were quite active at the all time points observed (Figure 2).

### Pulmonary pathology

**Figure 3:**
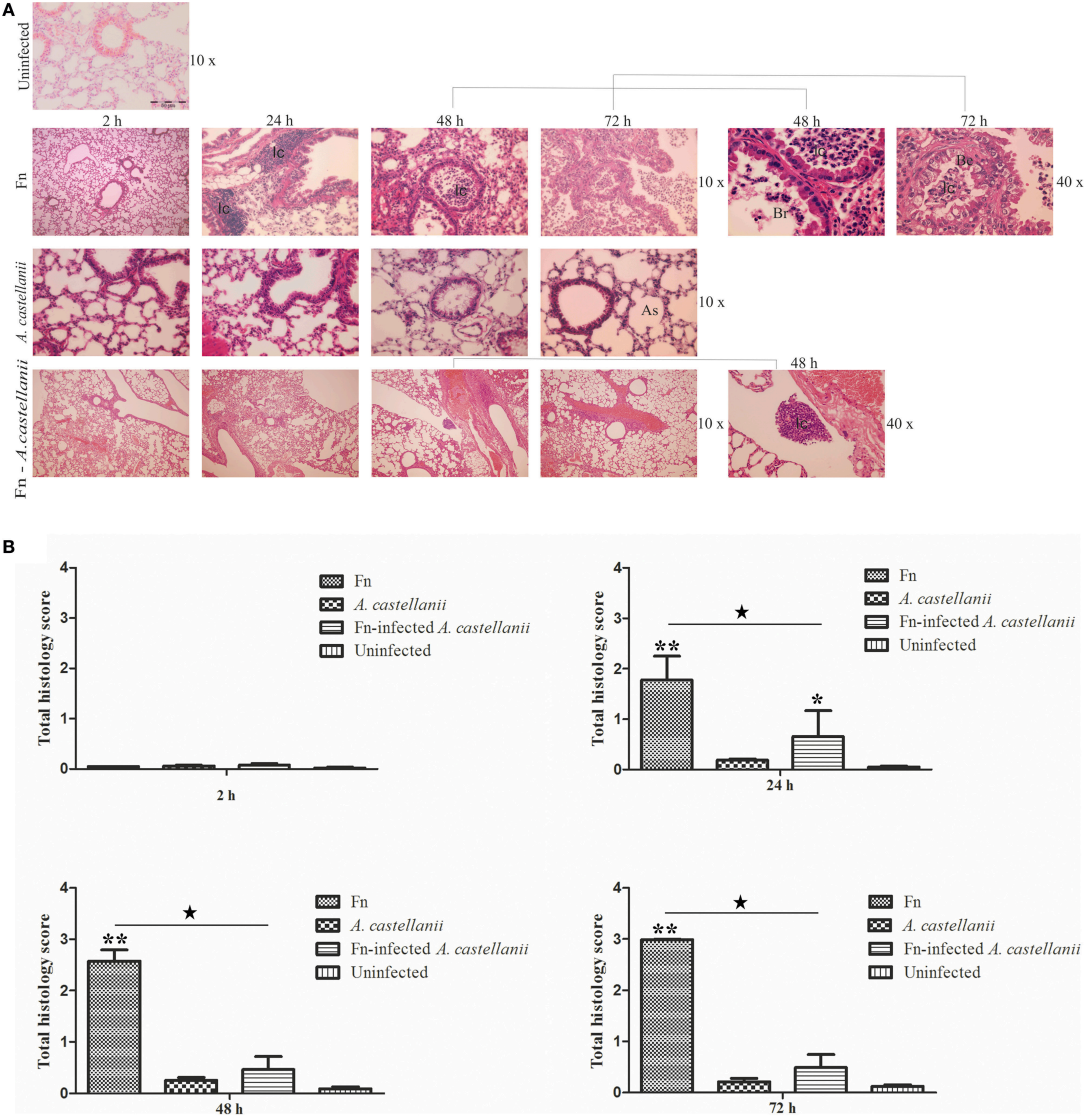
**Lung histopathology of Balb/c mice inoculated i.t. with *F. novicida*, *A. castellanii* and/or *F. novicida*-infected *A. castellanii*. (A)** At 2, 24, 48, and 72 h after infection of Balb/c, pulmonary tissue sections were stained with H&E (Hematoxylin and eosin stain). The experiments were done in triplicate using three mice for each time point, and the images are representative of 20 microscopic fields from each animal. Uninfected mice were used as negative control. The results are representative of three independent experiments. As-alveolar spaces, Br-bronchioles, Be-bronchial epithelium, Ic-inflammatory cells. **(B)** Twenty random high-powered fields (HPFs) were assessed to grade inflammation severity including alveolar and bronchial damage as well as percentage of parenchyma involved. The histology assessment included the number of the mononuclear cells and percent of parenchyma involved by using modification of double-blind scoring method at a magnification of 40x, as described previously (De Simone et al., 2014). The inflammation process was graded normal (score of 0) when there were 0–19 monocular cells infiltrates per HPF with no alveolar and bronchial involvement, mild (score of 1) for 20–49 cells per HPF including mild damage of alveolar and bronchial regions, moderate (score of 2) for 50–99 cells per HPF with moderate alveolar and bronchial inflammation, or severe (score of 3) for 100–200 mononuclear cells per HPF with severe effacement of alveolar and bronchial regions. The murine lung section was examined in sagittal direction and percent of parenchyma involved was scored as 0 when no area was compromised. The involvement of the parenchyma was scored as 1 when up to 25% of the total area was occupied by inflammatory exudate; was scored as 2 when 26 to 50% of parenchyma area was occupied with inflammatory cells, 3 if comprised more than 51%. The total histology score was calculated as an average of individual criteria scores. The uninfected tissue was used as a baseline score, ^*^P < 0.05 and ^**^P < 0.001. In addition, statistically significant differences between *F. novicida* and *F. novicida*-infected *A. castellanii* groups are marked by ^⋆^*p* < 0.05. The bar represents the median score in each group.

At the early time points of 2 h post infection in all examined groups the lungs of mice exhibited minor differences in terms of the cellular constituency (Figures 3A,B; *t*-test, *p* > 0.05). At 24 h after infection in the lungs of Balb/c mice infected only with *A. castellanii*, several mononuclear cells were present in peribronchial areas (Figures 3A,B). This cell type was generally absent from the lungs of uninfected mice even though was not statistically significant (Figures 3A,B; *t*-test, *p* > 0.05). At 24 h after infection with *F. novicida*, the pathological changes of the bronchiolar cells were characterized by a vacuolar degeneration (Figure 3A). The peribronchiolar spaces were infiltrated with mononuclear cells (Figure 3B; *t*-test, *p* < 0.001). The infiltration was also observed within bronchiole and alveoli but with less intensity than in peribronchiolar spaces (Figure 3A). In the group of mice inoculated with *F. novicida*-infected *A. castellanii* the infiltration process in pervivascular and peribronchial areas was present but with less extent then from the group of mice infected with *F. novicida* only (Figures 3A,B; *t*-test, *p* < 0.05).

At 48 and 72 h after infection the histopathological changes in the lung tissues of Balb/c mice infected with *A. castellanii* alone were minor (Figures 3A,B; *t*-test, *p* > 0.05). Some thickness of peribronchial walls was observed but was notably present in uninfected mice as well (Figure 3A). However, at 48 h after infection with *F. novicida*, in the lungs of Balb/c mice bronchopneumonia were present (Figure 3A). There were high numbers of inflammatory cells in the lumen of bronchia (Figures 3A,B; *t*-test, *p* < 0.001). The tissue surrounding the blood vessels were damaged and fibrinous exudates were present in alveolar spaces (Figure 3A). The histopatology changes at 72 h post infection with *F. novicida* alone were similar to those at 48 h after infection (Figure 3A). Surprisingly, the group of mice infected with *F. novicida*-infected *A. castellanii*, showed no signs of pulmonary pathology neither at 48 nor at 72 h of infection. The histopathology of these group of mice showed similar symptoms as group of mice infected with *A. castellanii* alone or the group of uninfected mice (Figures 3A,B; *t*-test, *p* > 0.05). The significant difference in overall histopathology score was found between group of mice infected with only *F. novicida* and *F. novicida*-infected *A. castellanii* mice (Figure 3B). Our results show that infection of mice with *F. novicida*-infected *A. castellanii* does not enhance the pulmonary pathology of mice.

### Inoculation of mice with *F. novicida*-infected *A. castellanii* blocks the recruitment of neuthrophils into mice lungs

It has been shown that neutophils are one of the first cell to be recruited in *Francisella* lung infection (Cowley and Elkins, 2011). We determined how *Francisella* infections impacted mononuclear and polymorphonuclear (PMN) cells populations in the lungs at 24 h after infection. We compared the influx of PMN cells, neutrophils, in the lung tissues of mice infected with *F. novicida*-infected *A. castellanii* in comparison to the lung tissues of mice infected with *F. novicida* and/or *A. castellanii* by flow cytometry.

**Figure 4:**
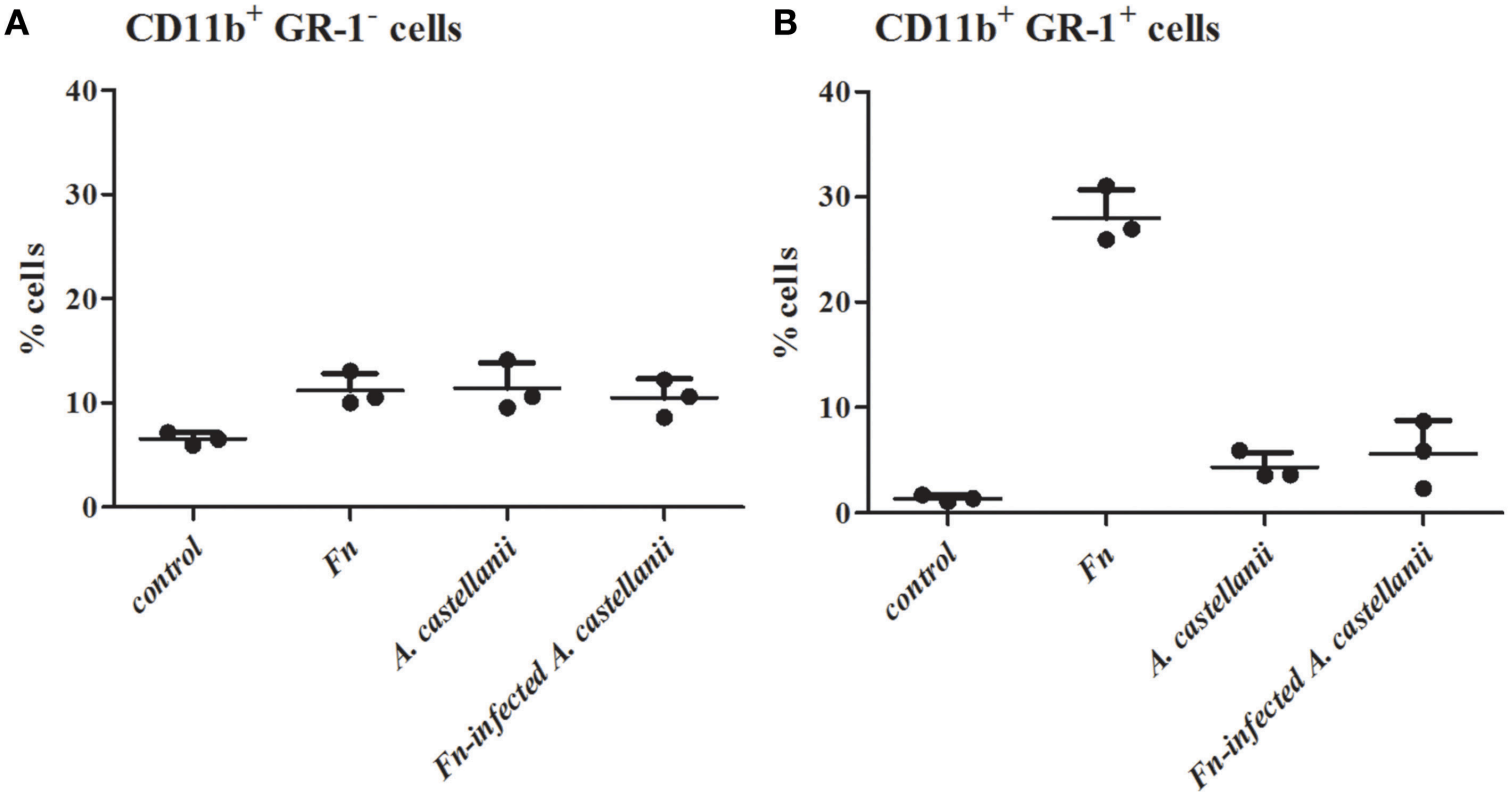
**Effect of *F. novicida*-infected *A. castellanii* on lung tissue macrophages (MNC, CD11b+ GR-1-) (A) and neutrophils (PMN, CD11b+ GR-1+) (B) cell recruitment into the lungs of Balb/c mice**. Balb/c mice were inoculated intratracheally with *F. novicida, A. castellanii* and/or *F. novicida*-infecetd *A. castellanii*. Uninfected mice were used as negative control. At 24 h after infection mice were sacrificed and inflammatory cell recruitment into the lungs was analyzed. Results represent the mean ± standard error of the mean of three animals per treatment group. Statistically significant differences between *F. novicida* and *F. novicida*-infected *A. castellanii* groups are marked by asterisk. ^*^p < 0.05.

Lungs from mice infected with *F. novicida, A. castellani* and/or *F. novicida*-infected *A. castellanii* had more CD11b^+^Gr1^−^ lung tissue macrophages then the lung of uninfected mice (*t*-test, *p* < 0.05; Figure 4A). Considering that mononuclear cells populations (CD11b^+^Gr1^−^) are very low at the day 1 after infection, it is possible that there is a balance between macrophage recruitment to the lung and macrophage killing.

A significant increase in the percentage of neutrophils (CD11b^+^Gr1^+^ cells) was evident at 24 h post infection in the lungs of mice infected only with *F. novicida* in comparison to uninfected mice (*t*-test, *p* < 0.05; Figure 4B). Consistent with previous data from this study there was no significant change in the percentage of CD11b^+^Gr1^+^ cells from *F. novicida*-infected *A. castellanii* mice compared to PBS control mice at 24 h after infection (*t*-test, *p* = 0.123). However, there is significant difference in percentage of neutrophils between the groups of mice infected with *F. novicida* and the group of mice coinfected with *F. novicida*-infected *A. castellanii* (*t*-test, *p* < 0.05). The percentage of neutrophils was slightly increased but not significantly in the groups of mice infected with *A. castellanii* in comparison to the group of uninfected mice (*t*-test, *p* = 0.087). There was no significant recruitment of neutrophils in the lungs of mice coinfected with *F. novicida*-infected *A. castellanii*.

### Vacuolar localization of bacteria in lung tissues of *F. novicida*-infected *A. castellanii*

It has been shown that the prior to bacterial escape into the cytosol of macrophages, where bacterial proliferation occurs, *Francisella*-containing vacuole (FCV) matures to a late endosome-like phagosome (Clemens et al., 2004; Santic et al., 2005a, 2008; Chong et al., 2008; Bröms et al., 2010; Asare and Kwaik, 2011). In mammalian cells the process of phagosomal disruption occurs within 30 min of infection (Golovliov et al., 2003; Checroun et al., 2006; Santic et al., 2010). In contrast, our previous *in vitro* studies showed that *F. novicida* does not escape from the vacuole in amoeba cells. The bacterium is enclosed within intact vacuole where it replicates (Santic et al., 2011). Based on the above findings we determined localization of the bacterium when inoculated with *F. novicida*-infected *A. castellanii* in the lung tissues of Balb/c mice.

**Figure 5:**
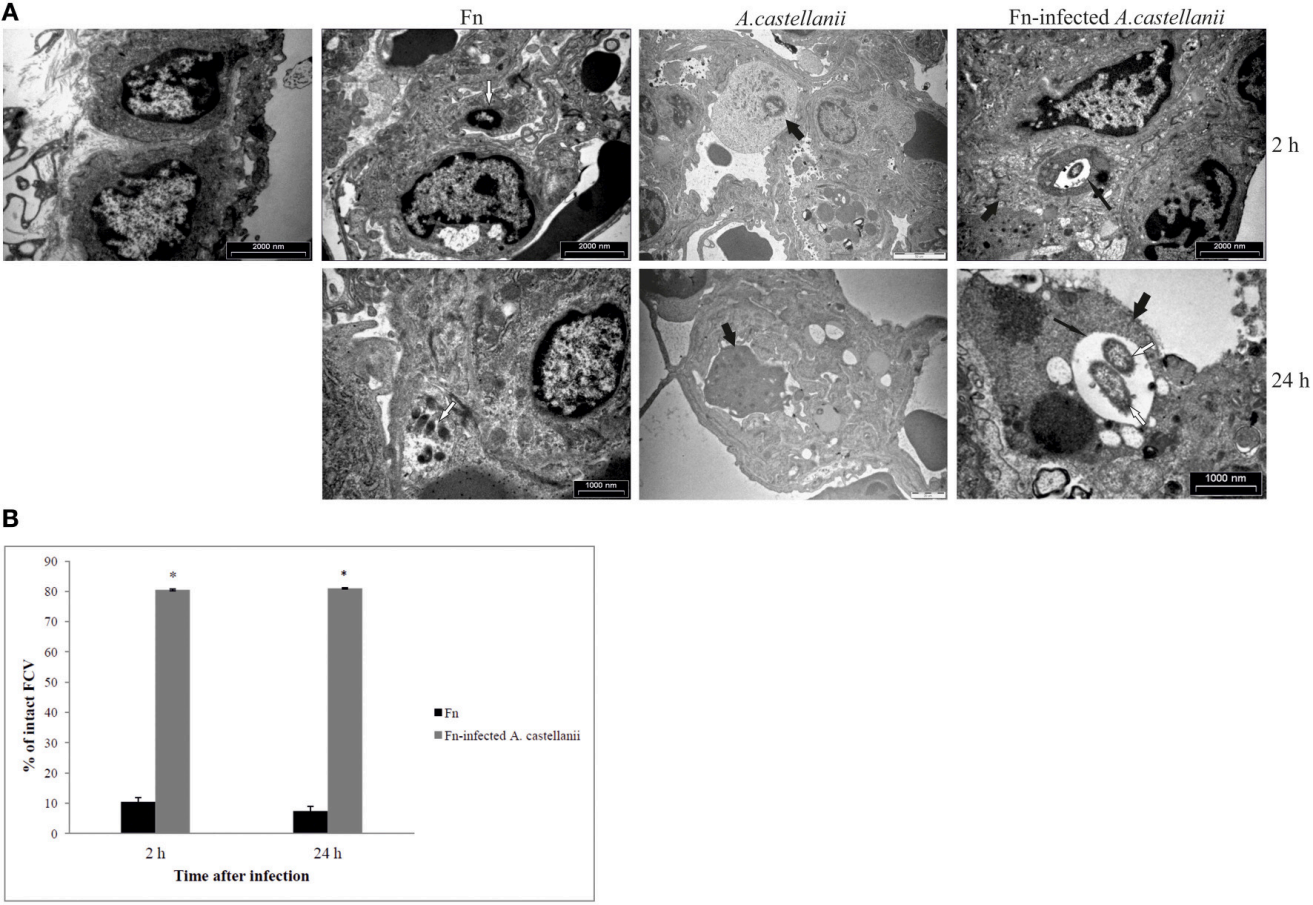
**Transmission electron micrographs of pulmonary tissues of Balb/c mice. (A)** At 2 and 24 h after infection of mice with *F. novicida, A. castellanii* and/or *F. novicida*-infected *A. castellanii* lungs were excised, fixed with glutaraldehyde and processed to electron microscopy. Uninfected mice were used as negative control. Thin black arrows show intact vacuolar membranes, tick black arrow show amoeba and white arrows show bacteria. **(B)** Quantitative analyses of the intact FCV (*Francisella* containing vacuole). The integrity of phagosomal membrane was determined by electron microscopy counting at least 100 bacteria for each sample and using following criteria: (a) cytosolic localization of bacteria, (b) vacuolar localization of bacteria- intact vacuoles. ^*^p < 0.05.

At 2 h after infection by *F. novicida*, the bacteria were present in the cytosol within alveolar macrophages in the lung tissues of Balb/c mice (Figures 5A,B). Only 10% of *F. novicida* were enclosed in intact vacuoles of infected alveolar macrophages in the lung tissue of Balb/c mice. In the lung tissue of mice infected with *F. novicida*-infected *A. castellanii* it was difficult to distinguish between alveolar macrophages and *A. castellanii* cells. The differences are determined based on ultrastrusture of the cells. In the group of *F. novicida*-infected *A.castellanii* mice, the bacteria were localized mainly within amoeba cells digested by macrophages. In this double phagocytosis 80 % of bacteria were within membrane bound vacuoles at 2 h after infection (*t*-test, *p* < 0.05; Figures 5A,B). The bacterium was also observed in the amoeba cells alone, and in the alveolar macrophages. Interestingly, when the bacteria were found in macrophage cells they were in the cytosol. In contrast, when the bacteria were found in the amoeba cells within the lung tissue they were found in vacuoles.

By 24 h after infection, the bacteria were replicating in alveolar macrophages of Balb/c mice. Around 8% of replicating *F. novicida* were localized in intact vacuoles (Figures 5A,B). In the experimental mice group of *F. novicida*-infected *A. castellanii*, the bacteria were localized in the vacuoles within amoebal cells in the lungs of Balb/c mice. Around 80% of *A. castellanii* harboring the bacteria had intact *Francisella*-containing vacuoles, in comparison to alveolar macrophages where bacteria were replicating in the cytosol (*t*-test, *p* < 0.05; Figures 5A,B).

## Discussion

Epidemiology of tularemia in some parts of the world, such as Sweden and Turkey, is associated with water-borne transmission, including mosquitoes and amoebae as the potential host reservoirs of the *Francisella* in water environment (Dai et al., 2010; Zogaj and Klose, 2010; Akimana and Kwaik, 2011; Asare and Kwaik, 2011; Broman et al., 2011; Gavrilin and Wewers, 2011; Jones et al., 2011; Simsek et al., 2012). It has been demonstrated that *F. novicida* and *F. philomiragia* can structure the biofilm *in vitro* (Durham-Colleran et al., 2010; Verhoeven et al., 2010). It has been hypothesized that *F. novicida* and LVS utilize *A. castellanii* as a natural reservoir (Abd et al., 2003; El-Etr et al., 2009; Santic et al., 2011). In addition, virulent strains of *F. tularensis* survive for weeks within *A. castellanii* (Abd et al., 2003; El-Etr et al., 2009).

Previous studies showed that amoeba-grown *L. pneumophilla* exhibited an increased capacity to enter tissue culture cells compared to agar-grown bacteria (Cirillo et al., 1994) and to be more infectious for mice (Cirillo et al., 1999). These results lead to the hypothesis that *L. pneumophila* that have been grown intracellulary in amoebae may be more infectious. *In vivo* studies have shown that as few as one inhaled or aspirated *L. pneumophila*-infected amoeba may constitute an infectious particle for Legionnaires' disease (Brieland et al., 1997a). In the environment, protozoa maintain *L. pneumophila* in natural aquatic and potable water systems, as they both provide a niche for bacterial replication and serve as a vehicle to protect *L. pneumophila* during the process of water treatment. We and others have previously demonstrated that Balb/c mice develop replicative *F. novicida* lung infection in response to intratracheal inoculation with virulent *F. novicida* cells (Santic et al., 2007b; Yu et al., 2008; Signarovitz et al., 2012). There is one study that shows BHI-grown *Francisella* cells closely mimic their host-adapted counterparts which have emerged from macrophages (Hazlett et al., 2008). The experiments conducted in parallel with BHI- or macrophage-grown *F. tularensis* cells indicate that these host-adapted bacteria induce very low levels of MAP kinase activity and stimulate little or no secretion of proinflammatory cytokines (Hazlett et al., 2008). It is not known whether inhalation of *F. novicida*-containing aerosols or microaspiration of contaminated water with amoeba could cause and enhance the disease, similar to *L. pneumophila* (Brieland et al., 1997a).

Results from this study shows that *F. novicida*-infected *A. castellanii* organisms do not induce a severe form of tularemia in Balb/c mice. In addition, *F. novicida* infected *A. castellanii* is even less pathogenic for mice than *F. novicida* alone. The intrapulmonary growth of *F. novicida* was significantly greater in mice inoculated with *F. novicida* alone compared to mice inoculated with *F. novicida*-infected *A. castellanii*, where there is inhibition of the bacterial growth. In addition, in contrast to systemic infection caused by infection of mice with *F. novicida* alone there was no such high dissemination of bacterium in the liver and spleen when mice were infected with *F. novicida* –infected *A. castellanii*. These findings are in contrast to *L. pneumophila* infections where *L. pneumophila* inhaled protozoa act as cofactors that enhance pathogenesis of Legionnaires' disease (Brieland et al., 1997a). It is possible that infection of alveolar macrophages during Legionnaires' disease imitate the intracellular infection of protozoa by *L. pneumophila*. The life style of *F. novicida* is very different in amoeba cells in comparison to mammalian cells, where cytoplasmic location of bacteria is crucial step in productive intracellular replication (Santic et al., 2011). In protozoa *Francisella* do not escape from the vacuole into the cytoplasm, but remain enclosed in vacuoles (Santic et al., 2011). It is possible that *F. novicida* is trapped within amoeba and the productive infection is not developed *in vivo.* This is consistent with a clinical study conducted in Sweden where water sampled in Ljusdal during 2004 contained high number of *F. tularensis* subsp. *holarctica* even though no human cases were recorded in the area (Broman et al., 2011).

Neutrophils are the important cells in controlling bacterial infections with a significant increased phagocytosis relative to macrophages (Kumar and Sharma, 2010). It has been shown that LVS strain of *Francisella* is quickly taken up by neutrophils, but the respiratory burst is prevented due to disruption of NADPH oxidase assembly within the phagosome (Allen and McCaffrey, 2007; Allen, 2013). In addition, recruitment of neutrophils and monocyte occurred in response to *Francisella* pulmonary infection, although these cells contribute to the progression of the disease by becoming host cells for *Francisella* replication (Hall et al., 2008). In contrast, inhalation of *F. novicida* did not lead to recruitment of neutrophils in the lungs after 4 h (Hajjar et al., 2006). Because *F. novicida*-infected *A. castellanii* did not potentiate intrapulmonary growth of *F. novicida* within 48 h postinoculation, we investigated the recruitment of inflammatory cells in the lung of infected Balb/c mice. Our results showed that the percentage of lung tissue macrophages were similarly present in mice inoculated with *F. novicida*-infected *A. castellanii* or inoculated with an equivalent number of bacteria. This suggests a proinflammatory effect of *A. castellanii* that is not related to *F. novicida* replication. In contrast, the percentage of neutrophil populations was much higher in the lungs of Balb/c mice being infected with *F. novicida* alone in comparison to *F. novicida*-infected *A. castellanii* and/or *A. castellanii* alone. In addition to neutrophil recruitment, we also observed a lower percentage of tissue macrophages in *F. novicida*-infected mouse lungs. There is speculation that *F. novicida* utilize neutrophils for replication more rapidly than they are being recruited to the site of infection. It is possible that the intravacuolar bacteria cannot stimulate macrophage, and thus do not trigger synthesis and secretion of cytokines that recruit neutrophils to the lungs during *F. novicida*-infected *A. castellanii.* It is possible that intravacuolar bacteria may block the immune response to recruit neutrophils to the lungs during *F. novicida*-infected *A. castellanii* infection.

In summary, our results demonstrate that in contrast to amoeba-infected by *L. pneumophila, F. novicida*-infected amoebae are not infectious particles in a murine model of tularemia and perhaps may not enhance bacterial virulence to humans.

## Author contributions

MO, MB, and MS contributed *in vivo* experiment and writing. IG and ZT participated in immunology part of experiment and writing. YA and VM participated in an *in vitro* experiment and writing.

## Funding

This work is supported by a University Grants (Grant No. 13.06.1.1.11 and 13.06.2.2.60). YA is supported by Public Health Service Award 1R01AI120244 and R21AI116517 from the National Institute of Health and by the Commonwealth of Kentucky Research Challenge Trust Fund.

### Conflict of interest statement

The authors declare that the research was conducted in the absence of any commercial or financial relationships that could be construed as a potential conflict of interest.
